# Artificial chemotaxis in micro/nanomotors

**DOI:** 10.1038/s41467-026-72137-w

**Published:** 2026-04-25

**Authors:** Roshan Velluvakandy, Xiaohui Ju, Martin Pumera

**Affiliations:** 1 Future Energy and Innovation Laboratory, Central European Institute of Technology, Brno University of Technology, Purkyňova 123, Brno, Czech Republic; 2 Advanced Nanorobots & Multiscale Robotics Laboratory, Faculty of Electrical Engineering and Computer Science, VSB - Technical University of Ostrava, 17. listopadu 2172/15, Ostrava, Czech Republic

**Keywords:** Actuators, Molecular machines and motors

## Abstract

Chemotaxis is the directional motion of objects in response to chemical gradients, a process that drives navigation in micro/nanomotors, enabling a bio-inspired transition toward autonomous direction control. However, current studies lack consistent mechanistic justification, definition, and standardized experimental validation. This review summarizes key physical principles governing active chemotaxis, surveys experimental strategies for generating chemical gradients and quantifying responses, and examines how individual chemotactic mechanisms and long-range, anisotropic chemical interactions give rise to emergent collective behaviors, while outlining prospective applications. Together, these efforts establish a principled engineering framework for advancing chemotaxis as a robust functional navigation modality in synthetic micro/nanomotors.

## Abstract (web summary)

This Review Article examines artificial chemotaxis in micro/nanomotors, detailing mechanisms, experimental strategies, and collective behaviors. It highlights the need for standardized validation and explores applications in biomedical and industrial fields, emphasizing the challenges and potential of synthetic chemotaxis.

## Introduction

Chemotaxis, the directed movement of cells or organisms in response to chemical gradients, is a fundamental biological process that governs a wide range of biological behaviors, from immune cell trafficking to bacterial foraging^1^. Since the pioneering work of Engelmann, Pfeffer, and Beijerinck in the late 1800s, our understanding of chemotaxis has expanded substantially, driven by numerous studies across microbiology, cell biology, and biophysics^2^. Early observations laid the foundation for unraveling the mechanisms by which both prokaryotic and eukaryotic cells sense the chemical cues in their environment^3^. The ability of living organisms to interpret and respond to these chemical cues with high specificity and efficiency has inspired researchers to replicate similar behavior in artificial systems. More recently, there have been attempts at engineering chemotaxis-like behavior in micro- and nanoscale particles more commonly referred to as micro/nanomotors^4–6^. These are synthetic systems engineered to move autonomously in fluidic environments at speeds exceeding those predicted by Brownian motion. This enhanced motion can be achieved through a variety of mechanisms; some micro/nanomotors are externally powered using light, magnetic fields, or ultrasound, where directional control is relatively straightforward, as it can be governed by modulating the applied fields. Others rely on self-generated propulsion driven by chemical reactions occurring on their surfaces^7^. In the case of these chemically powered micro/nanomotors, however, achieving directional control is far more challenging. While it is possible for such motors to navigate along chemical gradients, mimicking biological chemotaxis, they naturally lack the complex internal feedback or signaling mechanisms required for biological gradient sensing and steering.

Over the past decade, extensive research on artificial chemotaxis has shown that certain systems can exhibit spontaneous chemotactic behavior driven by asymmetric surface reactions, diffusiophoretic interactions, and related mechanisms^8–10^. In addition, there has also been steady progress in engineering chemotaxis in micro/nanomotors. However, despite steady progress, the field lacks a systematic overview of the underlying mechanisms, standardized experimental approaches, and a unified framework to guide the design and evaluation of artificial chemotaxis in micro/nanomotors. To bridge this gap, this review clarifies the distinction between active responses and passive transport phenomena. We begin by clarifying the terminology, strictly distinguishing between orthokinetic speed modulation and true vectorial taxis. Building on this, we analyze the scaling laws that differentiate the reorientation mechanisms of micron-scale motors from the ‘apparent chemotaxis’ often observed in nanoscale systems. We then expand the scope to collective dynamics, discussing how particle interactions and swarm behaviors can amplify sensitivity to weak gradients. Addressing the critical need for standardization, we evaluate the validity of current experimental setups and propose practices to rule out artifacts. Finally, we connect these fundamental principles to emerging biomedical and technological applications, highlighting the limitations that must be addressed to develop the next generation of chemotactic micro/nanomotors.

### On terminology: taxis and kinesis

**Fig. 1:**
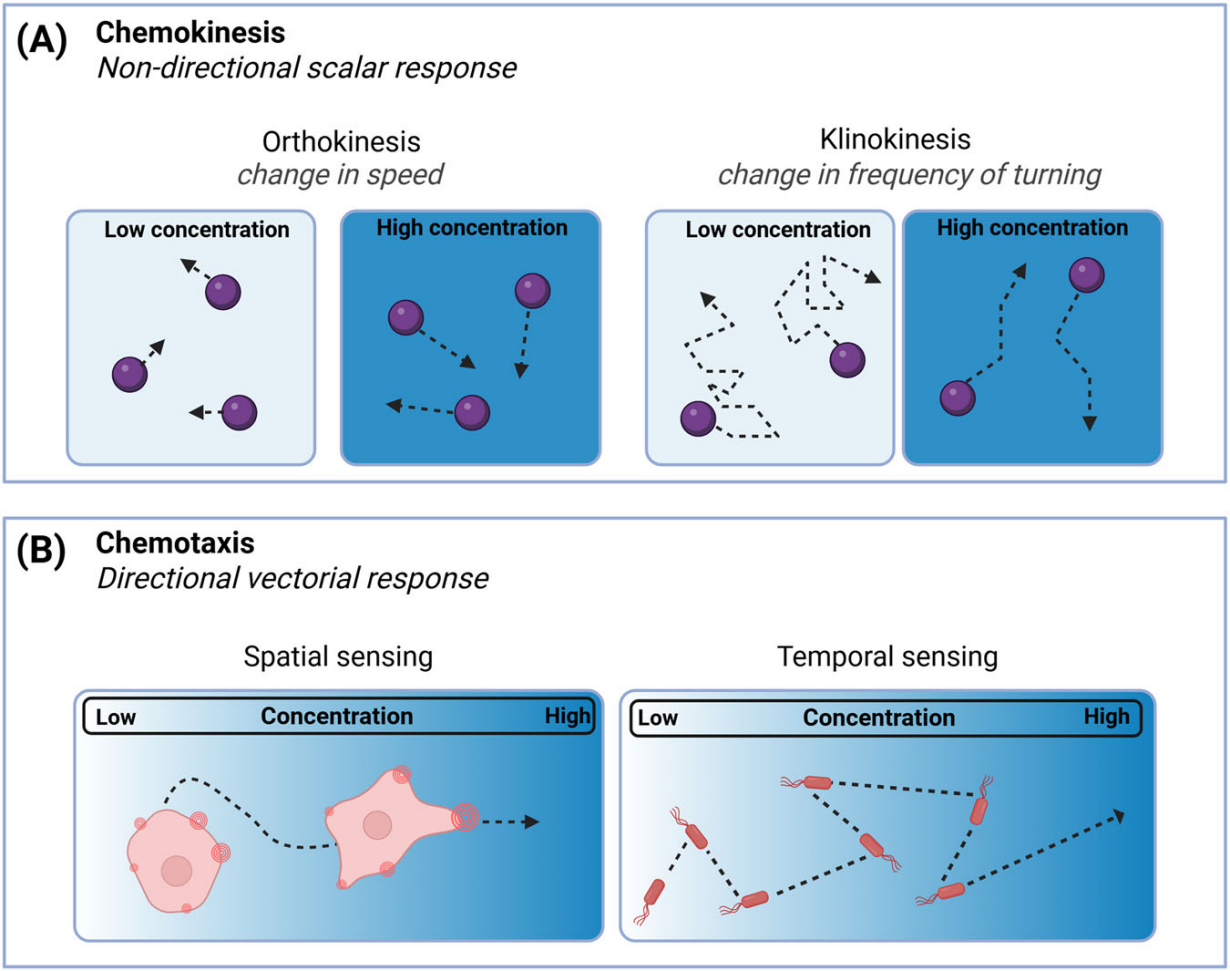
Terminology of chemically induced changes in orientation. **A** Chemokinesis, a non-directional, scalar response to a chemical stimulus. Particles/cells respond to concentration levels by altering their speed (orthokinesis) or the frequency of their turning (klinokinesis), resulting in random movement rather than directed motion. **B** Chemotaxis, a directional, vectorial response where movement is guided along a chemical gradient. Cells may navigate via spatial sensing, detecting concentration differences across the cell body simultaneously, or temporal sensing, comparing concentrations over time as the organism moves. Created in BioRender. Velluvakandy, R. (https://biorender.com/3k1m6qt).

To properly contextualize the specific mechanisms of micro/nanomotors, it is essential to first define chemotaxis and distinguish it from kinesis, as these are frequently conflated. Kinesis is a non-directional scalar response^11^, where chemokinesis, specifically, is a non-directional response where the intensity of a chemical stimulus determines certain parameters of motion rather than the direction^12^. This occurs in two main forms: orthokinesis, which involves a change in speed based on chemical stimulus intensity. For example, dendritic immune cells can increase their migration speed in response to inflammation signals. Klinokinesis, on the other hand, involves altering the frequency of turning events, as seen in *Escherichia coli*, which adjusts its random tumbling rate based on nutrient concentration without directly orienting its body axis towards the gradient (Fig. 1A). It is important to establish that in chemically powered micro/nanomotors, active motion is essentially kinesis; most motors exhibit positive orthokinesis and negative klinokinesis in response to their fuel.

Taxis, specifically chemotaxis, is a directional vectorial response where motion is oriented relative to a chemical gradient^12^. Classically, this implies spatial sensing and active reorientation of the body axis toward or away from the stimulus. However, modern definitions have evolved to include temporal sensing strategies, such as the biased 'run and tumble' behavior of *Escherichia coli* (Fig. 1B). Although *E. coli* employs a klinokinetic mechanism, modulating tumble frequency, it is classified as chemotactic because it uses temporal memory to bias a random walk into a net directional migration^1,13^.

For this review, we define chemotaxis in synthetic motors as self-powered active motion that acts directionally along a chemical concentration gradient, distinct from passive drift induced by the gradient. This directional bias may arise from active alignment or biased random trajectories. Crucially, particle accumulation alone does not confirm chemotaxis; accumulation can result from passive trapping or chemokinetic slowing. Therefore, distinguishing active directional steering from passive retention is vital.

### Fundamental transport mechanisms in solute gradients

To study chemotaxis in micro/nanomotors, it is first necessary to decouple the active responses of chemotaxis from the fundamental transport mechanisms that affect any particle in a concentration gradient. A solute gradient breaks the spatial symmetry of the local thermodynamic environment. For suspended particles, this asymmetry manifests physically as phoretic transport; these processes generate directed motion purely through physical force balances and exist independently of the particle’s capacity for self-propulsion.

The most prominent example of these mechanisms is diffusiophoresis, the migration of a colloidal particle in response to a concentration gradient of a solute that interacts with the particle surface. This phenomenon relies on a thin interfacial interaction layer where the local potential energy between the solute and the particle surface differs from the bulk. When the solute is non-ionic, such as glucose, the interaction is governed by short-range forces like van der Waals or steric repulsion. The concentration gradient establishes a pressure gradient within this interaction layer parallel to the particle surface, driving a slip velocity that propels the particle. In the case of electrolyte gradients, the physics is more complex and comprises two contributions: chemiphoresis, which arises from the pressure imbalance within the electric double layer, and a macroscopic electrophoretic term. The latter occurs because ions with different diffusion coefficients generate a spontaneous macroscopic electric field to maintain electroneutrality, which subsequently acts on the charged particle to induce drift. The combination of these effects typically results in much faster migration speeds for electrolytes compared to neutral solutes due to the long-range nature of electrostatic forces^14^.

A unique case arises with semi-permeable particles in a gradient of impermeable solutes, differences in osmotic pressure across the particle boundary cause the particle to expel water from the side facing higher pressure and admit water on the opposing side. This osmophoretic flow drives the particle toward the low-solute concentration region, effectively mimicking negative chemotaxis^15^.

Phoretic or convective drift imposes a velocity vector on the particle externally; the particle is thermodynamically “pushed” or “pulled” by the field regardless of its orientation. Crucially, these phoretic effects are purely passive responses to external gradients and do not constitute chemotaxis. An active motor exhibits chemotaxis only if it actively biases its otherwise random trajectory relative to the gradient.

### Chemotactic and kinetic responses in micro/nanomotors

With the fundamental physics of phoresis established, we now examine how micro/nanomotors utilize these forces to interact with chemical gradients. Historically, the distinction between simple speed modulation (kinesis) and true directional motion (taxis) was often blurred. We begin by addressing the historical context surrounding chemokinetic accumulation before defining the strict requirements for chemotaxis and contrasting these with passive phoretic drift.

### Orthokinesis in micro/nanomotors

**Fig. 2:**
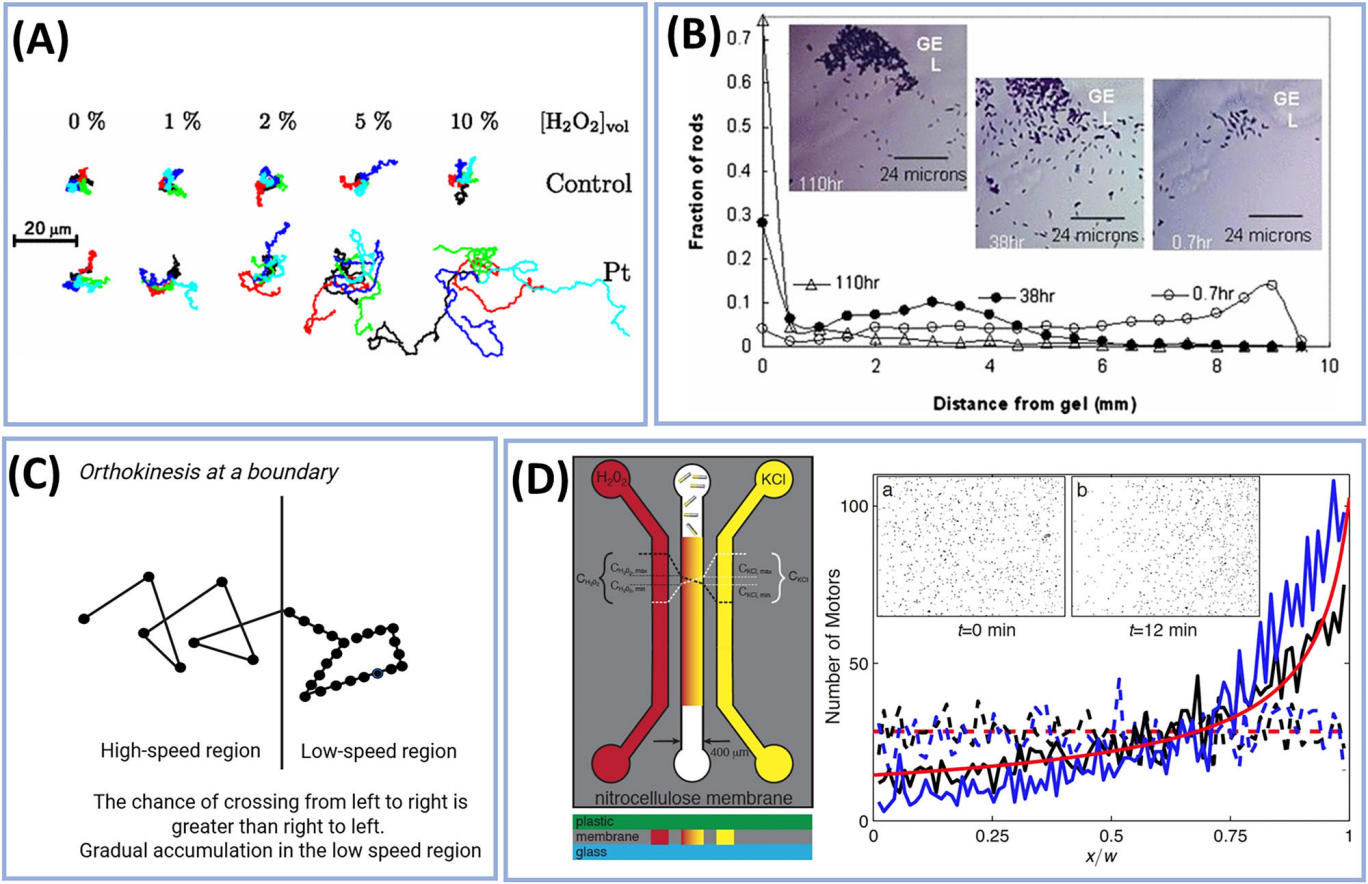
Orthokinesis in micromotors. **A** Trajectories of Pt-PS Janus micromotors showing positive orthokinesis; linear diffusivity increases and rotational diffusion decreases as fuel (H_2_O_2_) concentration increases. Reprinted with permission from ref. ^17^. Copyright 2007 American Physical Society. **B** Early observations of Au/Pt nanorods accumulating near a high-concentration hydrogel source over 110 hours, initially suggesting chemotaxis. However, this contradicts expectations with positive orthokinesis, which predicts accumulation in low-speed regions for these motors. Reprinted with permission from ref. ^18^. Copyright 2007 American Physical Society. **C** Description of positive orthokinesis at a boundary. Particles moving randomly will statistically accumulate in the low-speed region because it is easier to enter the slow zone from the fast zone than to escape it. Adapted with permission from ref. ^19^. Copyright 1985 Elsevier. (**D**) Validation of orthokinetic accumulation of nanorods in a controlled gradient showing accumulation in low-mobility regions rather than high-fuel regions, confirming that without active reorientation, positive orthokinesis leads to accumulation in slow zones. Adapted from ref. ^16^. CC BY 4.0.

In the case of chemically powered micro/nanomotors, most of the reported cases exhibit primarily positive orthokinesis to their own fuel, where the speed of the motor increases with fuel concentration, and in some cases, negative klinokinesis, where the rate of turning, or frequency of change in direction, decreases as the fuel concentration increases, leading to longer straight runs^16^. This feature of ortho- and klinokinesis led to one of the earliest suggestions for how artificial chemotaxis could be realized in synthetic micro/nanomotors^17^. This was based on the observation that the speed of self-electrophoretic platinum-polystyrene (Pt-PS) Janus micromotors increases with increasing concentration of fuel (H_2_O_2_ in this case), while their rotational diffusion time decreases, scaling inversely with the fuel concentration (Fig. 2A). Based on these observations, Howse et al. proposed the possibility of designing a system in which the linear and rotational diffusivities of a particle could be independently controlled, potentially enabling a mechanism analogous to the ‘run’ and ‘tumble’ temporal chemotaxis observed in bacteria^17^.

Based on the chemotaxis theory put forth by Howse et al. in 2007, Hong et al. explored the chemotactic behavior of bimetallic platinum/gold (Pt/Au) nanorods in the vicinity of a hydrogel soaked with H_2_O_2_ as a fuel. They observed the rods accumulated near the fuel source over a period of several days, leading to inferences of chemotactic behavior of the Janus nanorods. However, this observation contrasts with what would be expected from a primarily orthokinetic particle, which typically exhibits increased speed in response to higher fuel concentrations without any directional bias (Fig. 2B)^18^. With positive orthokinesis, particles or cells in a concentration gradient would accumulate in the regions where they are the slowest, *i.e*., the regions of low fuel concentration. To further elaborate, we consider the biological context demonstrated by Wilkinson’s experiment, where neutrophils were placed in a field divided by a boundary separating high- and low-speed regions^19^. The neutrophils accumulated predominantly in the low-speed region due to orthokinesis (Fig. 2C). This occurs because cells in the fast-speed region can easily cross the boundary to enter the slow-speed region, but once there, they have a lower statistical possibility to return to the fast-speed side, leading to their accumulation in the slow-speed region.

This unexpected observation of positive chemotaxis with Pt/Au nanorods by Hong et al. was subsequently investigated by Byun et al., who proposed that advective flows generated by the hydrogel source could account for the behavior^20^. In addition, the authors also developed a helpful framework to distinguish between self-phoretic and advective forces that might contribute to particle motion^20^. As a common experimental setup for studying chemotaxis, the same group published a detailed study highlighting the influence of advective flows from hydrogel sources and provided practical guidelines to improve future experimental designs^21^. In 2021, Moran et al. further demonstrated the chemokinetic, or more specifically, orthokinetic behavior of Pt/Au nanorods, using a microfluidic device that generated concentration gradients of H_2_O_2_ and salt, which influences the speed of the micromotors^16^. In such a device, the nanorods accumulated preferentially in the low mobility/high salt regions as predicted by orthokinesis (Fig. 2D).

In principle, all chemically powered micro/nanomotors exhibiting positive orthokinesis to their fuel are expected to accumulate in low fuel-concentration regions. For instance, researchers demonstrated with phototactic micromotors that when the particle’s re-orientation rate does not respond with the gradient, the micromotors do not exhibit taxis toward higher concentration regions^22^. Without active reorientation to align micro/nanomotors with the gradient, or a distinct process that suppresses the motion in high fuel-concentration areas, true artificial chemotaxis toward a high fuel-concentration gradient is unlikely to occur.

### Reorientation-based chemotaxis in micromotors

**Fig. 3:**
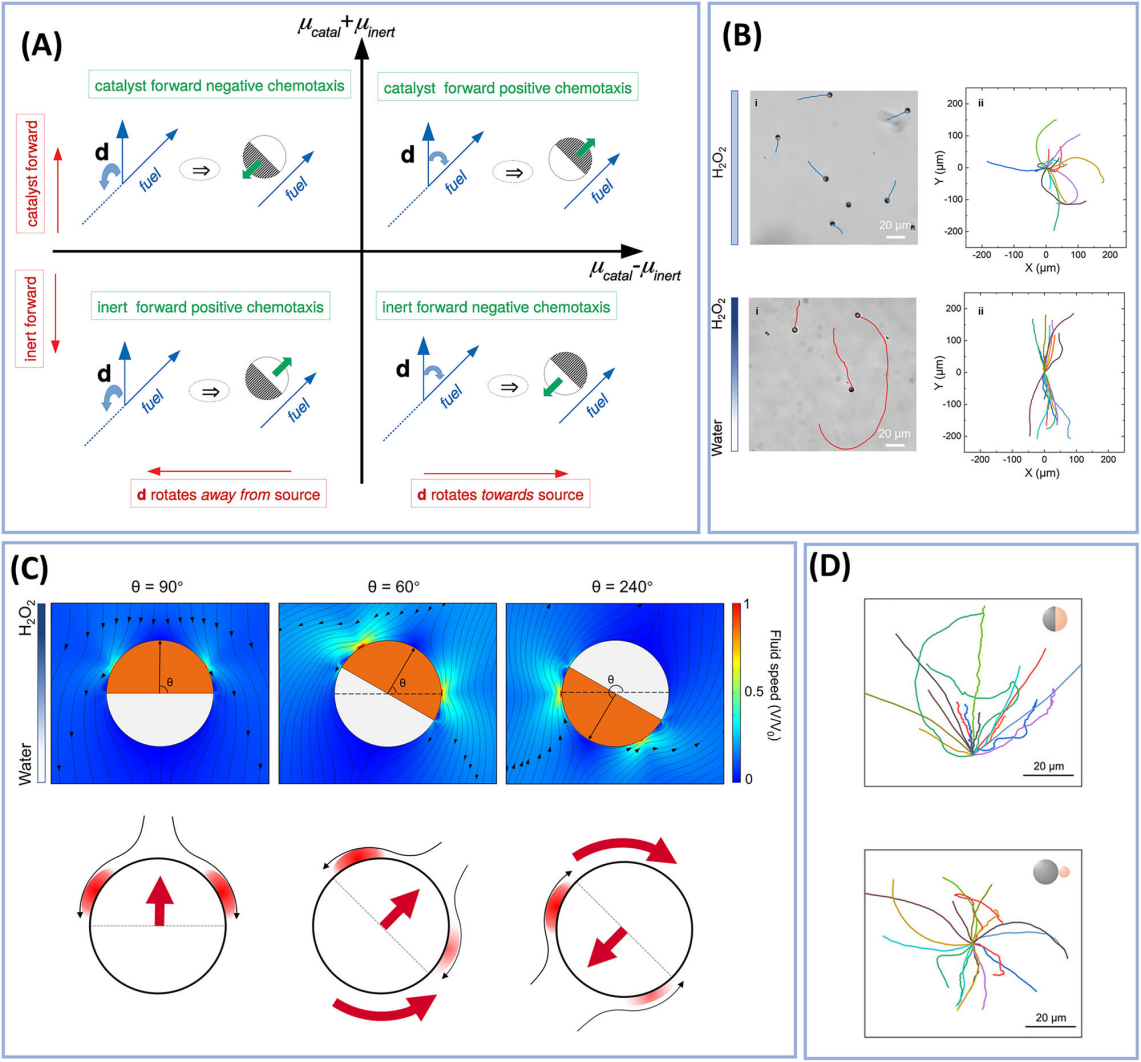
Reorientation-based chemotaxis in micromotors. **A** Phase diagram proposed by Popescu et al. categorizing the chemotactic behavior of Janus particles. The direction of the phoretic torque and linear motion depends on the contrast between the phoretic mobilities of the catalytic and inert faces, leading to either positive or negative chemotaxis. Reprinted with permission from ref. ^26^. Copyright 2018 American Chemical Society. **B** Experimental observation of chemotaxis in Cu/SiO_2_ micromotors in an H_2_O_2_ gradient. The trajectories reveal a coexistence of positive and negative chemotactic behaviors. Reprinted with permission from ref. ^28^. Copyright 2022 Wiley-VCH. **C** Hydrodynamic simulations quantifying the reorientation torque. When the particle axis is misaligned with the concentration gradient, unbalanced osmotic flows generate a restoring torque that aligns the particle with the gradient. Reprinted with permission from ref. ^28^. Copyright 2022 Wiley-VCH. **D** The influence of structural asymmetry on chemotaxis. Trajectories of Ag/SiO_2_ doublets demonstrate that reducing the size of the inert sphere diminishes the chemotactic response, confirming that distinct asymmetry is essential for generating reorientation torques. Reprinted from ref. ^30^. CC BY 4.0.

Following Hong et al., subsequent studies investigated the reorientation mechanisms driving artificial chemotaxis. In a pioneering work, Baraban et al. compared the behavior of catalytic Pt/SiO_2_ Janus spheres and tubular microjets, both powered by catalyzing H_2_O_2_ decomposition^23^. Using a Ψ-shaped microfluidic channel to generate a transverse H_2_O_2_ gradient, they observed that both motor architectures actively reoriented towards higher fuel concentrations. Critically, Janus spheres demonstrated superior reorientation capabilities compared to tubular jets, with the chemotactic response intensifying at higher fuel concentrations. While exceptions exist, such as manganese oxide–silica “matchstick” motors, the majority of subsequent research has prioritized chemotaxis studies with Janus-type asymmetry^24,25^. Popescu et al. proposed that a Janus particle undergoes rotation in response to a chemical gradient, if there is a variation in phoretic mobility across its surface^26^. This rotation arises due to phoretic slip flows generated along the particle surface because of concentration differences of solute molecules. The direction and strength of these flows depend on the material properties and their interactions with the surrounding solutes, collectively described by the phoretic mobility. Depending on the contrast between the mobilities of the catalytic and inert faces, the particle tends to reorient either toward or away from the gradient, enabling a positive or negative chemotactic response (Fig. 3A)^26^.

One of the first experimental validations of this reorientation-based mechanism was demonstrated by Mou et al. using ZnO-SiO_2_ Janus micromotors^8^. These utilized CO_2_ as fuel, reacting to generate ionic gradients of zinc and carbonate ions that power electrolyte self-diffusiophoresis. When the active ZnO cap was misaligned with the CO_2_ source, the difference in reaction rates and surface potentials across the Janus body created unbalanced electro-osmotic flows. These flows generated a restoring torque that continuously steered the swimmer back towards the gradient source^8^.

To isolate reorientation behavior from flow-related effects like the cross-stream effect in microfluidic channels, where active particles near a confining surface may align perpendicular to the flow direction and migrate across streamlines^27^, Xiao et al. developed a microfluidic stop-flow setup^28^. Here, a linear concentration gradient is first formed under flow, after which the flow is halted using a retroactive loop that equalizes pressure across the system. With such a stop-flow setup, the authors studied Cu-SiO_2_ micromotors in a gradient of H_2_O_2_ as fuel. They observed that the micromotors moved directionally, either toward higher or lower fuel concentrations (Fig. 3B). However, more micromotors biased their orientation toward the high-fuel concentration region. In principle, they identify two main cases. In the first case, when the axis of the particle is aligned with the concentration gradient, either towards the high or low concentration, in such a case, the particle is unaffected by torque and keeps its orientation. In the second case, when the axis of the particle is misaligned with the gradient, the torque reorients the particle to align with the gradient toward the high concentration region (Fig. 3C). This observation well aligns with the design principle proposed by Popescu et al. that particles with their catalytic cap facing the fuel move faster than those oriented the other way. This speed difference creates a bias, causing more particles to move toward the higher concentration region^26,28^. The dependence of the motion direction on the phoretic mobilities, as theorized by Popescu et al., was further verified by the same group using SiO_2_-Pt Janus particles. In this case, chemically modifying the silica hemisphere with either positively or negatively charged functional groups can reverse the direction of micromotor motion. Negatively charged SiO_2_-Pt micromotors exhibited predominantly negative chemotaxis, while the positively charged, amine-modified SiO_2_-Pt micromotors moved in the opposite direction. This provides direct experimental evidence that controlling surface charge, and thereby the phoretic mobility, can fundamentally alter artificial chemotactic behavior and lead to tunable direction control^29^.

Physical geometry plays an equally pivotal role. The fundamental requirement of structural asymmetry for effective reorientation was recently demonstrated experimentally with doublet Ag-SiO_2_ micromotors^30^. In these studies, decreasing the size of the inert SiO_2_ sphere relative to the active silver sphere, effectively reducing the extent of symmetry breaking, led to a significantly lowered extent of chemotaxis. (Fig. 3D). This confirms that distinct geometric anisotropy is essential for generating the necessary differential flows that drive the reorientation torque.

The reorientation mechanism is governed by both the surface phoretic mobility and the structural asymmetry, together these allow for the generation of a phoretic torque which allows a micromotor to steer in chemical gradients. Critically, however, this strategy relies on the particle’s ability to sense a gradient across its own body length and maintain a stable orientation against external forces. As explored in the following section, these requirements confront severe physical limitations at the nanoscale, necessitating a fundamental re-evaluation of how directed transport is achieved when rotational Brownian motion begins to dominate hydrodynamic torques.

### Phoretic drift and directional migration in micro/nanomotors

Effective reorientation-based chemotaxis requires a particle to sense a concentration difference across its body length and generate a torque strong enough to overcome Brownian rotation. However, as the particle size decreases to nanoscale regime, two critical issues arise: firstly, the concentration gradient across the particle becomes negligible; secondly, the rotational diffusion coefficient increases drastically. These constraints in nanoscale motors prohibit the reorientation-based chemotaxis observed in micron-sized Janus motors.

To bypass spatial limits, biological systems, like bacteria, often utilize temporal sensing akin to a biased 'run and tumble'. Yet, this mechanism faces a distinct barrier in synthetic particles, which is the requirement for an internal memory. The particle must compare the current concentration with a past value to determine if it is moving up-gradient. Without this temporal integration, concentration-dependent motility results merely in chemokinesis rather than taxis leading to accumulation in low mobility regions.

Consequently, many directional behaviors observed at this scale likely do not represent true chemotaxis through active directional bias, but rather a form of “apparent chemotaxis” or “pseudochemotaxis”, a phenomenon where the particle undergoes a net directional migration due to asymmetric phoretic drift, diffusion biases, or other mechanisms that operate without actively locking its orientation toward the source.

**Fig. 4:**
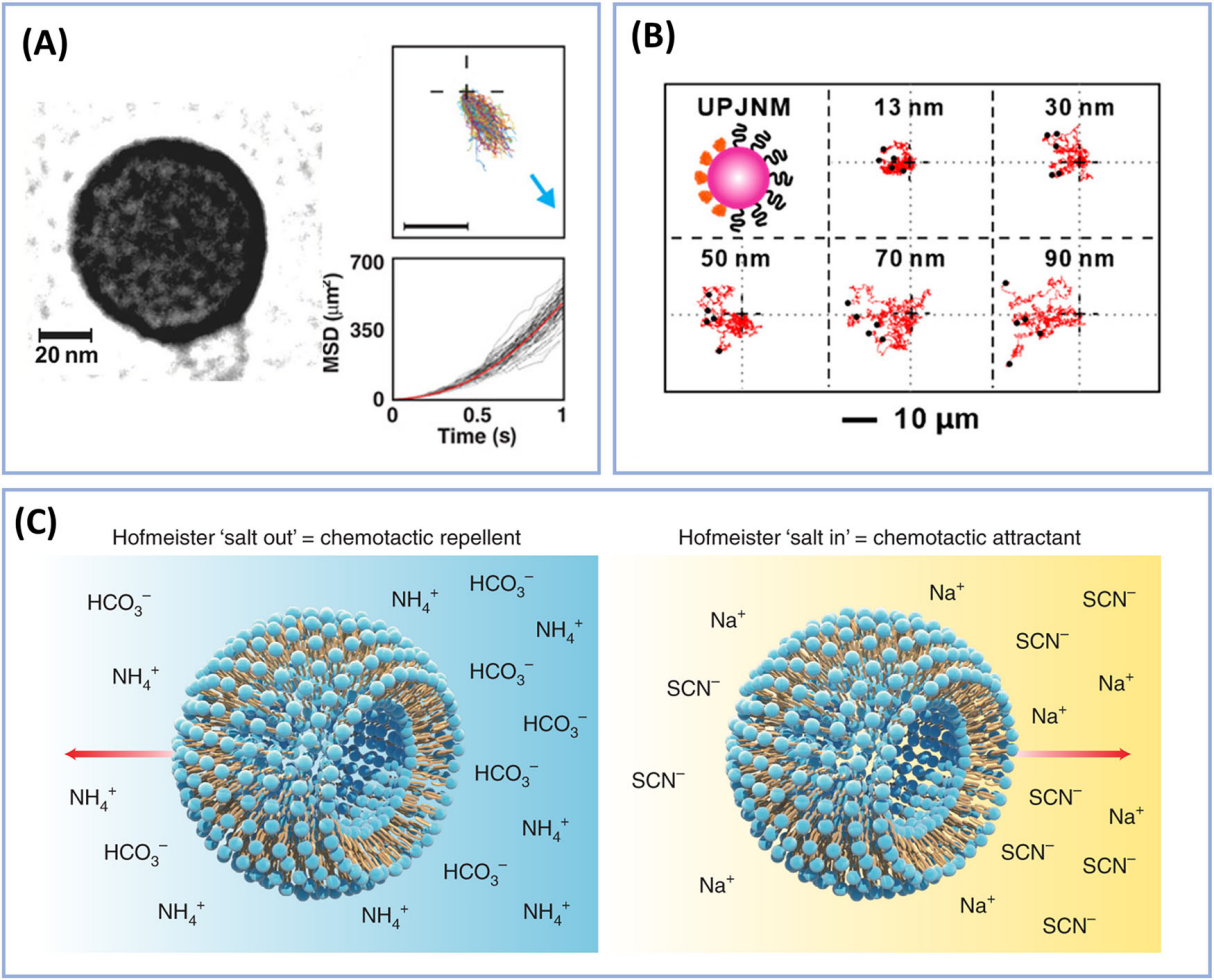
Phoretic drift and directional migration in nanomotors. **A** TEM image and trajectories of 50 nm enzyme-loaded polymersome motors. These nanomotors were observed to exhibit directional migration in glucose gradients. Adapted from ref. ^9^. CC BY 4.0. **B** Directional motion in ultrasmall urease-powered nanomotors. Trajectories for particles ranging from 13 nm to 90 nm show that directional bias persists even at the smallest scales. Reprinted with permission from ref. ^32^. Copyright 2023 American Chemical Society. In both (**A**) and (**B**), directional motion is likely a result of phoretic drift or potentially advection. **C** The Hofmeister effect as a driver of chemotaxis. Enzyme-coated liposomes are repelled by their own hydrolysis products leading to negative chemotaxis. The direction of drift is determined by the salting-out or salting-in nature of the generated ions, creating a gradient defined by the lyotropic series. Reprinted with permission from ref. ^84^. Copyright 2019 Springer Nature.

Numerous studies have reported directional migration in sub-micron systems, down to the scale of single enzymes^9,10,31–34^. With a few exceptions^10^, the fundamental basis of apparent nanoscale chemotaxis remains unclear in most works. For submicron motors, with sizes in the range of a few hundred nanometers^31,35–41^, for instance, 800 nm ‘flask like’ motors^37^ or smaller stomatocyte motors 300 nm^31^, depending on the specific architecture, reorientation-based chemotaxis may still be possible. However, observing this reorientation through microscopic methods at such scales becomes a challenge as they get closer to the diffraction limit. For the most part, reports on chemotaxis in nanoscale motors^9,10,32–34,42–45^, such as 50 nm enzyme loaded polymersome motors^9^ or 13 nm urease coated particles^32^, involve either tracking individual particle trajectories, without the ability to judge the orientation or studying chemotaxis at a collective level with microfluidic systems where a collective bias to a direction or accumulation are taken as signs of chemotaxis. (Fig. 4A, B). These methods are detailed in a later section, yet diffusiophoretic drift remains the most likely driver of the apparent chemotaxis observed in these works.

Another contributing mechanism to the apparent chemotaxis in soft nanomotors arises from the Hofmeister effect^10^. This effect describes the classification of ions based on their ability to “salt-out” (aggregate) or “salt-in” (solubilize) biomolecules, a phenomenon dictated by the ion’s size, shape, and hydration thermodynamics. Somasundar et al. demonstrated that enzyme-coated 100 nm liposomes can exhibit negative chemotaxis driven by the Hofmeister effect. (Fig. 4C). This behavior was consistent across different catalytic systems, where both urease-coated and ATPase-bound liposomes in their respective substrate gradients migrated away from their respective well-hydrated hydrolysis products, ammonium carbonate and ADP/phosphate. In both cases, the directional drift is driven by the enzymatic generation of these specific charged species, which create a repulsive gradient defined by the lyotropic series and drive the liposomes away to lower concentration regions^10^.

Perhaps the most intriguing case is the apparent chemotaxis exhibited by single, freely diffusing enzymes in gradient of their substrates. In pioneering experiments, Sengupta et al. showed that enzymes (catalase and urease), drift up their respective fuel gradients^33^. They also found that enzyme diffusion increased with substrate concentration, *e.g*., urease diffused faster in higher urea concentrations.

However, enhanced diffusion of enzymes remains a controversial topic with several opposing reports, succinctly reviewed by Feng and Gilson^41^. Controversies exist for observations of chemotaxis in enzymes as well with opposing observations of positive or negative chemotaxis for specific enzymes^33,46^. However, the phenomenon itself is well accepted and recent work has reconciled several of these discrepancies^47^.

To explain the physical basis of these observations, several theoretical frameworks have been proposed^47–49^. Agudo-Canalejo et al.^47^ proposed that the apparent chemotaxis in enzymes is governed by the competition between nonspecific diffusiophoresis and specific binding-induced diffusion enhancement. They propose that while diffusiophoresis generally drives enzymes toward higher substrate concentrations, binding-induced enhanced diffusion creates a drift toward lower concentrations. The direction of transport is therefore determined by substrate concentration, where phoretic effects dominate above a critical threshold, and binding-induced mechanisms prevail at lower concentrations. This model resolves previously reported discrepancies in urease apparent chemotaxis, reconciling early observations by Sengupta et al. of positive chemotaxis in high concentrations of urea^33^, with later reports by Jee et al.^46^ obtained at lower concentrations that indicated negative chemotaxis. In practice, most enzymes studied show net positive chemotaxis. Zhao et al. demonstrate that each enzyme in a glycolytic cascade, including hexokinase, phosphoglucose isomerase, phosphofructokinase, and aldolase, independently migrated up its specific substrate gradient. This substrate-driven assembly of enzymes shows that each enzyme “follows” the chemical trail of its substrate^50^.

Thus, distinct from the active reorientation seen in large micromotors, current nanoscale transport is defined by the physical laws of diffusiophoresis and thermodynamic interactions. Yet, this reliance on phoretic drift is likely a temporary constraint. The existence of bacterial temporal sensing proves that overcoming Brownian rotation to achieve directional bias is physically feasible even for small entities. The integration of such temporal logic into synthetic designs represents the next evolutionary step, potentially enabling nanomotors to move beyond apparent chemotaxis toward true, active gradient tracking. Until such individual sophistication is achieved, however, significant directional behaviors often emerge not from the lone swimmer, but from the statistical dynamics of the population. This brings us to the domain of collective chemotaxis, where inter-particle interactions and ensemble responses give rise to macroscopic directionality.

### Collective behavior driven by chemotaxis

Having established that individual micro/nanomotors can chemotactically navigate in external chemical gradients, we now consider the collective regime where these gradients may not always be imposed, but self-generated. In suspensions of micro/nanomotors, every particle may simultaneously act as a source of chemical gradients and at the same time respond to these gradients.

**Fig. 5:**
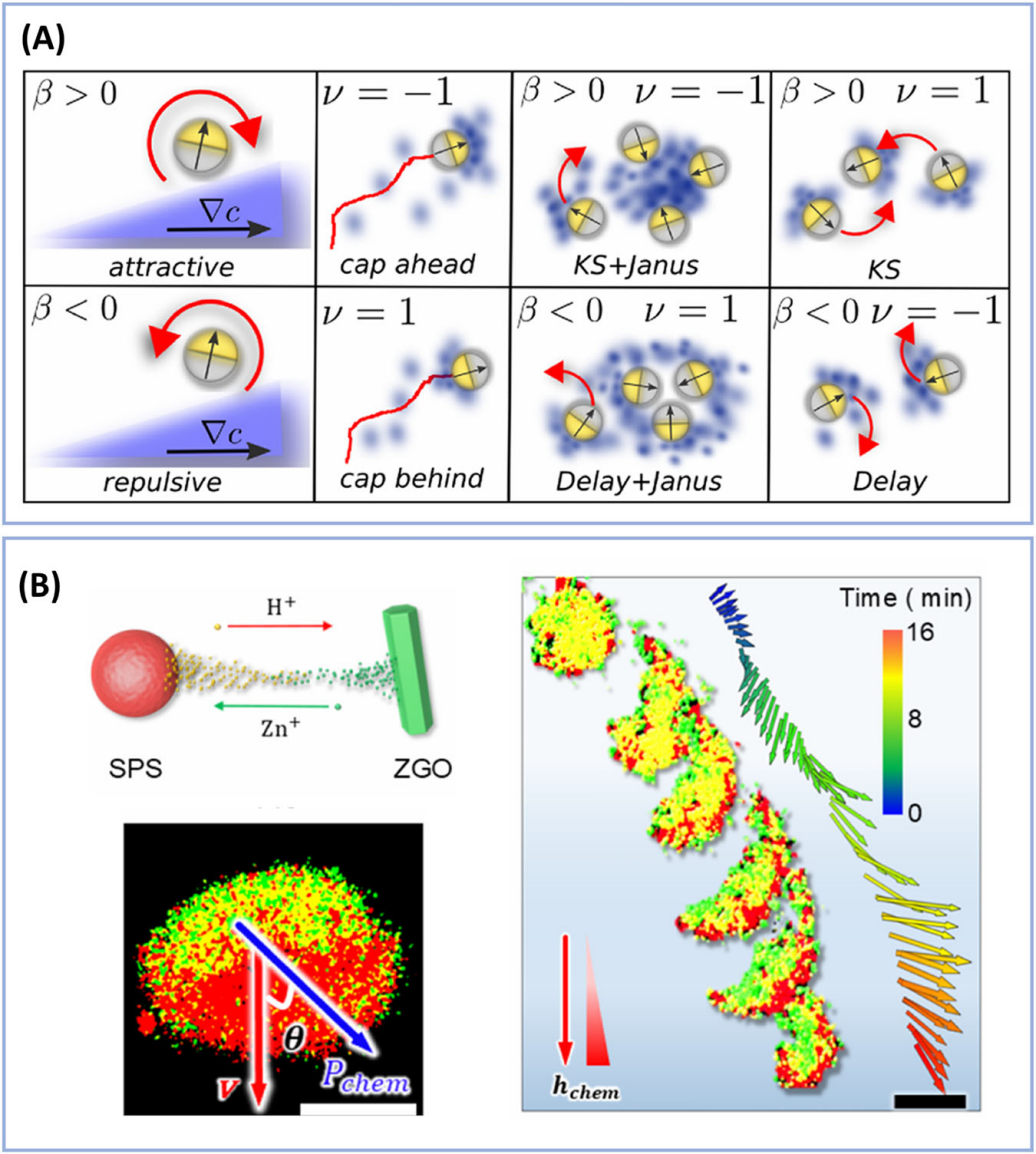
Collective behavior driven by chemotaxis. **A** Collective behaviors predicted by the Phoretic Brownian Particle model. The behavior is governed by the chemical interaction parameter (*β*) and the alignment coupling (*ν*). Attractive interactions (${{{\rm{\beta }}}} > \,0$) lead to Keller-Segel (denoted as KS) collapse or dynamic clustering. Repulsive interactions (${{{\rm{\beta }}}} < \,0$) also generate structures through Janus instability (denoted as Janus), where clusters are stabilized by self-trapping in repulsive shells or delay instability (denoted as Delay) where oscillatory patterns are caused by delayed response to chemical fluctuations. Reprinted with permission from ref. ^51^. Copyright 2017 American Physical Society. **B** Macroscopic ferrochemical chemotaxis in a non-reciprocal swarm. A mixture of ZGO nanorods and SPS microbeads spontaneously phase-separates due to ion-exchange interactions. This forms a soft Janus cluster with a macroscopic chemical dipole. The swarm actively rotates and steers toward proton sources, amplifying weak external gradients. Adapted with permission from ref. ^61^. Copyright 2025 American Chemical Society.

Their collective dynamics are best described by the Phoretic Brownian Particle (PBP) model (Fig. 5A), which explicitly accounts for the coupling between self-propulsion, reorientation, and chemical signaling^51^.

In this framework, the position ${r}_{i}$ of the $i$-th particle changes as it swims with a constant self-propulsion velocity ${v}_{0}$ in a direction given by the unit vector ${{{{\bf{p}}}}}_{i}=\left(\cos {\theta }_{i}(t),\sin {\theta }_{i}(t)\right):$^52^

1 $$\dot{{{{{\bf{r}}}}}_{{{{\rm{i}}}}}}={v}_{0}{{{{\bf{p}}}}}_{i}$$

The particle’s swimming direction ${\theta }_{i}$ is not fixed as it reorients in response to the chemical gradient. This is described by the Langevin equation for the orientation^52^

2 $$\dot{{\theta }_{i}}=\beta {{{{\bf{p}}}}}_{i}\times \nabla c\left({{{{\bf{r}}}}}_{i}\right)+\sqrt{2{D}_{r}}{\xi }_{i}\left(t\right)$$

Here, $\beta$ is the alignment coupling coefficient, which acts as a torque strength: if $\beta > \,0$, the particle turns toward the gradient (attraction), whereas if $\beta < \,0$, it turns away (repulsion). This rotation competes with random rotational diffusion, characterized by the coefficient ${D}_{r}$ and Gaussian white noise ${\xi }_{i}\left(t\right)$.

Finally, the chemical field evolution must account for the specific geometry of the Janus particles. The PBP model uses a source term that integrates over the particle surface^52^

3 $$\dot c ({{{\bf{r}}}},t)={D}_{c}{\nabla }^{2}c({{{\bf{r}}}},t)-{k}_{{d}}{c}({{{\bf{r}}}},t)+\mathop{\sum }_{(i=1)}^{N}\oint d{{{{\bf{x}}}}}_{i}\delta ({{{\bf{r}}}}-{{{{\bf{r}}}}}_{i}(t)-{R}_{0}{{{{\bf{x}}}}}_{i})\sigma ({{{{\bf{x}}}}}_{i})$$

This integral is taken over the surface of particles with radius ${R}_{0}$. The function $\sigma \left({{{{\bf{x}}}}}_{i}\right)$ represents the non-uniform production rate density, which is non-zero only on the catalytic hemisphere of the Janus colloid^51,52^.

When the interactions are attractive, particles swim toward self-generated chemical maxima as in the Keller-Segel instability, however, once a critical density is exceeded, active systems often exhibit dynamic clustering. In the dynamic clustering regime, clusters constantly exchange particles with the dilute gas phase. The cluster size is self-limited because the outward swim pressure of the active particles competes with the inward phoretic attraction. If the phoretic attraction is strong enough, the system can undergo a full collapse; however, moderate activity often stabilizes finite-sized, fluctuating clusters that merge and breakup, a state distinct from the static clusters seen in equilibrium phase separation.

Perhaps the most counter-intuitive insight from the PBP model is that chemorepulsion, also drives robust pattern formation. One might expect repulsion to simply homogenize the system, but Liebchen et al.^51,53^ identified two generic instability mechanisms that lead to structure. The first is Janus instability which arises from anisotropic chemical production. Since Janus particles produce chemicals only on their catalytic hemisphere, a cluster of particles pointing inward creates a chemical peak at its center. If the particles are chemorepulsive, they are trapped in this configuration by the shell of chemicals they produce, stabilizing a cluster of finite size. This size scales linearly with the propulsion speed. The second case is a delay-induced instability which occurs when a uniform chemical field undergoes a weak fluctuation, chemorepulsive colloids naturally cluster in the field’s minima. These colloids generate chemicals that oppose the fluctuation, but due to a finite response delay, they fail to stop at equilibrium. Instead, they overshoot the target, inverting the chemical profile. This over-correction can amplify the disturbance, resulting in an oscillatory instability^51–53^.

Experimental evidence for chemorepulsive organization is found in active emulsions. Hokmabad et al.^54^ demonstrated ‘chemotactic self-caging’, where swimming droplets are transiently trapped by the repulsive chemical trails of their neighbors. Unlike crowding in glassy systems, this history-dependent caging occurs at very low area fractions because the chemical trails persist and diffuse long after a particle has passed.

A unique instance of collective behavior arises when two chemically distinct passive particles are mixed, leading to non-reciprocal interactions^55^. In such systems, a particle of species A may be attracted to the chemical trail of species B, while species B is simultaneously repelled by A. Theoretically, this frustration prevents equilibrium assembly and leads to an unbound chasing regime where the “predator” pursues the “prey”^56^. This creates active molecules^55^ or self-propelling dipoles that continuously convert chemical energy into translation. This dynamic was experimentally demonstrated by Meredith et al*.*^57^ using mixtures of chemically active, immiscible oil droplets, where non-reciprocal surface tension modulation caused predator droplets to chase prey droplets. Similar behavior was observed in solid-state systems of TiO_2_ and ZnO particles^58^, and using ion-exchange resins^59,60^. The significance of these microscopic non-reciprocal interactions culminates in their ability to drive macroscopic “ferrochemical” behavior. Wu et al.^61^ demonstrated that a mixture of zinc germanate (ZGO) nanorods and sulfonated polystyrene (SPS) microbeads can self-organize into a macroscopic, dynamic swimmer (Fig. 5B). Through non-reciprocal ion exchange, these swarms spontaneously phase-separate to form a soft Janus asymmetry, characterized by a distinct separation between ZGO-rich and SPS-rich poles. This separation creates a massive macroscopic chemical dipole across the entire cluster. When exposed to an external proton gradient, the swarm rotates its internal polarization axis to align with the chemical field, actively steering the collective assembly toward the proton source. Because the cluster is macroscopic and dynamic, it can amplify weak external chemical gradients by over 10^4^ times, significantly higher than individual motors. This giant amplification enables the swarm to robustly track weak signals, such as the acidic microenvironment of dental biofilms, making it one of the most chemically sensitive and robust examples of artificial chemotaxis to date^61^.

### Alternative chemotaxis strategies

Apart from phoretic mechanisms, directional transport can also be achieved through interfacial tension gradients or by co-opting biological swimmers. The Marangoni effect, driven by surface tension differences, enables “droplet swimmers”^62–66^ such as decanol droplets in salt gradients^62^, to navigate complex paths and solve mazes. While visually similar to biological taxis, these systems are typically limited to two-dimensional interfaces and sizes well above the micro/nanoscale. Alternatively, biohybrid systems bypass synthetic limitations by attaching cargo to naturally chemotactic cells^67,68^. These semi-synthetic architectures leverage biological chemotaxis for sensing, utilizing examples such as sperm cells^69^ or magnetotactic bacteria, which perform magneto-aerotaxis to navigate oxygen gradients^70^. However, as these systems rely on biological chemotaxis rather than synthetic mechanisms, they lie outside the primary scope of this review.

### Experimental frameworks for evaluating chemotaxis and recommended practices

The reliability of chemotaxis observations relies heavily on the interplay between the physical properties of the micro/nanomotors and the fidelity of the chemical environment. As experimental designs evolve from simple diffusion assays to complex microfluidic architectures, it becomes critical to distinguish genuine tactic behavior from environmental artifacts. This section outlines the methodologies for establishing gradients and the requisite controls to ensure data reliability.

### Physical characterization and pre-requisites

Before evaluating chemotactic performance, a rigorous assessment of the micro/nanomotors physicochemical characterization is essential. Subtle variations in surface chemistry, particle charge, or structural asymmetry can drastically alter active motion and reorientation dynamics, often leading to contradictory results across comparable studies. For chemotaxis, the surface charge is a governing parameter^26,29^, consequently, the zeta potential must be quantified under experimentally relevant conditions. This is particularly nuanced for Janus particles as the two faces possess distinct zeta potentials. True values of the faces can be estimated by synthesizing and characterizing homogeneous particles of each material separately. However, it is the overall, mean zeta potential, measured with methods such as dynamic light scattering, that effectively acts as the governing parameter for phoretic transport. Crucially, if surface modifications such as fluorophore labeling are employed for tracking, characterization should be performed post-modification to ensure the tactic potential remains unaltered. Similarly, for Janus motors, the extent of asymmetry affects both active motion and chemotaxis^30^. While physical deposition methods like sputtering and atomic layer deposition inherently produce visible asymmetry in micromotors, chemically synthesized and nanoscale motors require validation via mapping or advanced imaging techniques to confirm the anisotropic distribution necessary for active steering.

### Generation of concentration gradients

#### Classical diffusion assays

**Fig. 6:**
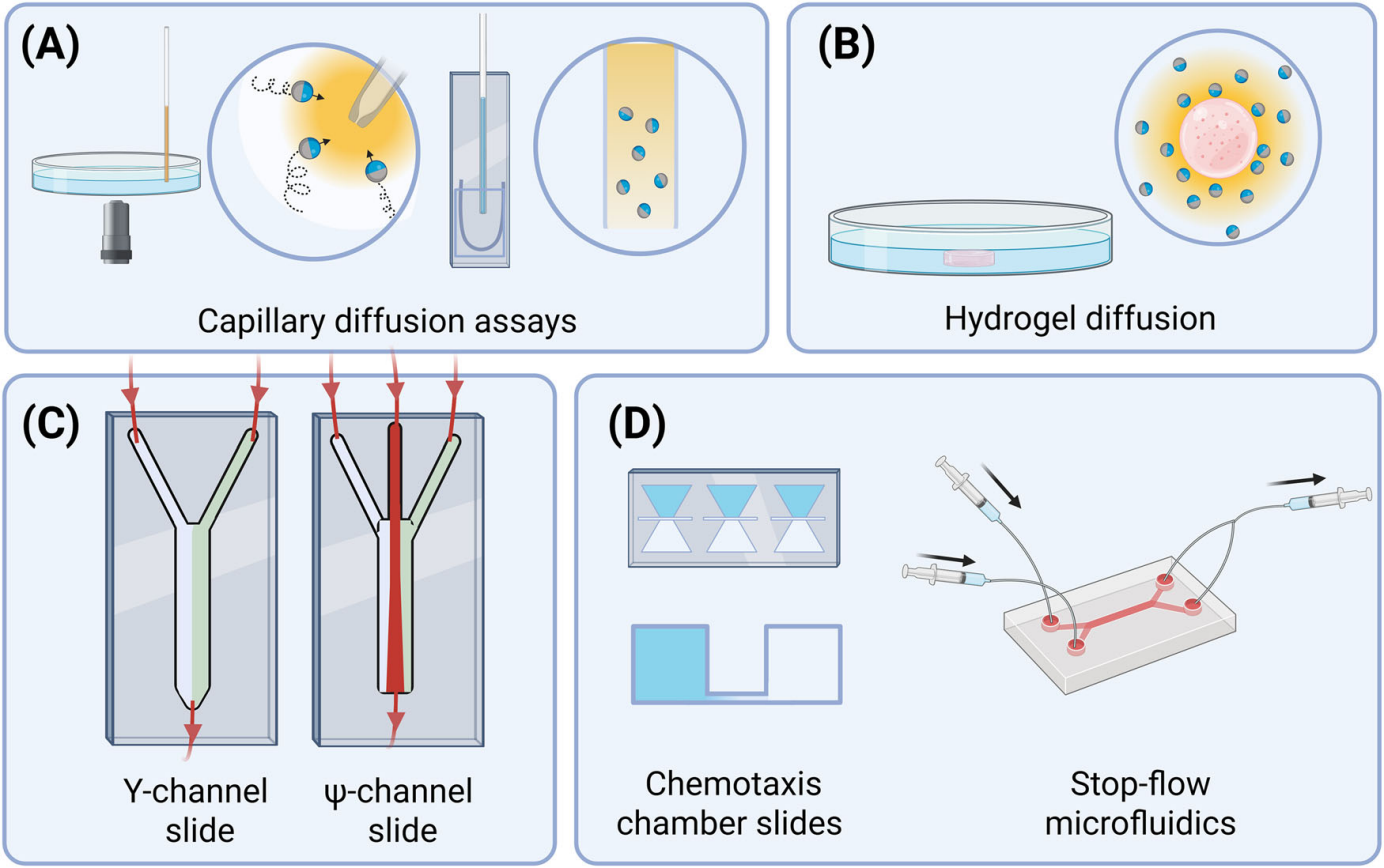
Experimental platforms for generating concentration gradients. **A** Capillary diffusion assays: a classical method where a fuel-filled capillary creates a gradient at the interface. It is susceptible to physical trapping artifacts such as the capillary effect, independent of chemotaxis. **B** Hydrogel diffusion: a simple method where a hydrogel block releases chemoattractant. Although widely used, it requires strict control of the hydrogel block and chamber geometry to prevent density-driven advective flows that can mimic directed motion. **C** Continuous-flow microfluidics: Y-channel and Ψ-channel setups utilize laminar flow to create diffusion interfaces. These allow for observations of positive or negative interactions but can lead to cross-stream effects and do not distinguish phoretic drift from chemotaxis. **D** Static microfluidic systems: commercial chemotaxis slides (e.g., Ibidi µ-slides), classic chemotaxis slides such as Zigmond or Dunn chambers and complex systems such as stop flow microfluidics, which can create stable gradients while minimizing advective flow. Created in BioRender. Velluvakandy, R. (https://BioRender.com/yj72kqn).

The most classical approach in studying chemotaxis involves static diffusion, primarily via capillary or hydrogel assays. The capillary diffusion assay, a derivative of Pfeffer’s bacterial assay^71^, utilizes a fuel-filled capillary to generate a steep, yet predictable gradient at the interface with the bulk medium (Fig. 6A)^8,18^. While capable of maintaining gradients for extended periods, up to an hour for small molecules like methylene blue, the method is susceptible to significant artifacts. Solovev et al. demonstrated that capillary forces can act as a physical trap, drawing microjets into the tube independent of chemotaxis^72^. Thus, data from capillary assays must be scrutinized to decouple chemotaxis from physical capillary forces. As a result of these inherent drawbacks, the application of the capillary assay has seen a marked decline over time.

Similarly, hydrogel plug assays offer a straightforward platform where an agar or similar hydrogel block releases chemoattractant into the surrounding medium contained in a small vessel (Fig. 6B). The setup itself and its variants are one of the most commonly used methods for evaluating chemotaxis to date^9,18,31^, although advantageous in its ease of use and allowance of direct microscopic observation, this method frequently introduces advective flow^20^. As the hydrogel swells or absorbs water, it generates localized flows that can passively drag particles toward the source. Recent guidelines by Sapre et al. highlight that strictly controlling hydrogel density, moisture content, and chamber geometry is necessary to dampen these advective effects^21^. Specifically, they recommend using chamber heights below 250 µm and ensuring hydrogels are thoroughly presoaked, sometimes for up to 24 hours, to minimize density-driven convection^21^. In both capillary and hydrogel setups, the inclusion of control experiments involving passive tracer particles is critical and allows to differentiate capillary or advective forces from chemotaxis^45^. Ultimately, while the capillary assay has largely fallen out of favor, hydrogel-based methods retain significant utility due to their simplicity. However, the field is increasingly witnessing a shift toward microfluidic methods, which offer superior control over gradient stability and fluid dynamics.

#### Microfluidic approaches

Microfluidics provides superior control over gradient spatiotemporal profiles compared to bulk methods. Initial studies frequently employed continuous-flow systems, such as the three-inlet Ψ-type or two-inlet Y-type channels (Fig. 6C)^23,33^. These setups utilize laminar flow to create sharp diffusion interfaces, allowing for the observation of particle reorientation and migration orthogonal to the flow direction. While these dynamic systems enable the simultaneous screening of positive and negative behaviors^10^, they are susceptible to the cross-stream effect where active particles near a surface tend to align perpendicular to the flow direction and also create transverse motion^27^. Although this limits much of their practical applicability in studying genuine chemotaxis, flow-based channels remain valuable for diffusiophoresis studies^73^, where they serve as an effective platform for qualitatively determining the attractive or repulsive nature of a solute with respect to the motor. However, due to the confounding interplay of phoresis and this cross-stream migration, the field is increasingly moving toward static microfluidic environments to ensure accurate performance assessment.

One of the first static gradient generation methods employed in this context was utilized by Joseph et al., who adapted the commercial Nanosight (Malvern Panalytical) system^9^. Since this platform leverages laser-based dark-field scattering microscopy to enable the direct, label-free tracking of particles below the diffraction limit, it has become a widely used tool in nanomotor tracking studies^74–76^. However, the Nanosight observation chamber is relatively large compared to dedicated microfluidic channels; at high solute concentrations, this volume becomes prone to significant convective and advective flows^77^. These bulk fluid movements can overshadow subtle tactic motion, making it imperative to validate gradients in such large-volume chambers against passive drift controls^45^.

One of the most robust methods for evaluating chemotaxis is the “stop-flow” protocol introduced by Xiao et al.^28^. In this approach, a gradient is initially established under continuous flow before a retroactive pressure loop stops the flow, permitting observation in a quiescent environment (Fig. 6D). This technique effectively eliminates cross-stream drift while preserving gradient linearity. However, this method necessitates a moderately complex experimental setup to achieve precise hydraulic balancing. As a simpler alternative, commercial platforms such as Ibidi µ-slides^45^ or classical Dunn and Zigmond chambers offer standardized, low-height geometries that suppress convection and maintain stable gradients over long periods^45,77,78^. These static configurations are generally preferred for quantitative analysis, as they minimize the complex hydrodynamic coupling inherent to flow-based tracking.

Despite the enhanced stability offered by these static microfluidic platforms, experimental parameters must still be rigorously tuned to prevent artifacts. Specifically, the use of excessive solute concentrations can reintroduce density-driven advection^77^, destabilizing the quiescent gradients these systems are designed to preserve. Furthermore, as the field advances toward biomedical implementation, it is critical to align experimental conditions with physiological realities. Chemotactic assays should prioritize biologically relevant concentration gradients rather than arbitrarily steep ones, ensuring that the observed active transport accurately reflects the motor’s potential behavior within the constraints of a realistic in vivo environment.

### Necessary controls for chemotaxis validation

**Fig. 7:**
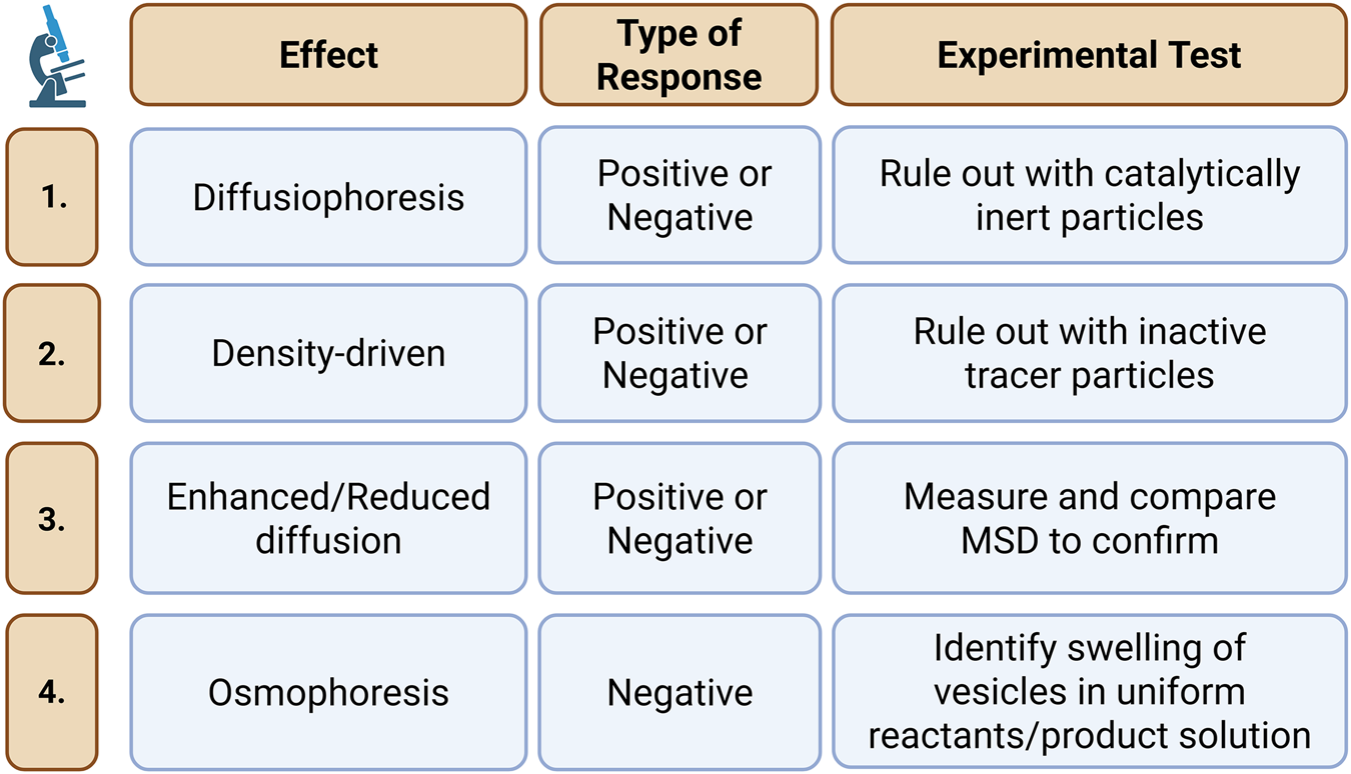
Common physical effects that mimic chemotaxis. The table summarizes artifacts that must be ruled out to confirm active steering. **1** Diffusiophoresis is driven by external passive solute-surface interactions rather than active motion; if catalytically inert analogs migrate, the motion is passive. **2** Density-driven advection occurs when steep gradients, due to high concentration of solute or chamber geometry, create convective flows that drag particles, a phenomenon confirmed if passive tracers share the motor’s trajectory. **3** Enhanced diffusion can lead to particle accumulation in specific regions due to changes in fuel availability, but unlike chemotaxis, it lacks directional persistence or angular bias. **4** Osmophoresis results from an imbalance in solvent pressure due to solute absorption or exclusion, often evidenced by the physical swelling of vesicles. Created in BioRender. Velluvakandy, R. (https://BioRender.com/qr324e1).

Establishing genuine chemotaxis requires a rigorous experimental design that explicitly decouples active steering from passive transport phenomena (Fig. 7). Since observed directional bias is frequently a result of artifacts like phoresis or advection, a robust study must rely on a tiered system of controls. Firstly, to rule out advection, the simultaneous tracking of passive tracers is necessary. If inert beads match the motor’s trajectory, the motion is undoubtedly advective. Similarly, non-catalytic analogs, such as enzyme-free particles with identical surface chemistry, must be tested to isolate passive solute-surface interactions. If these inactive controls migrate up-gradient, the driving force is likely diffusiophoretic drift rather than active steering.

Crucially, for micromotors, validation must go beyond velocity data by explicitly evaluating the particle’s reorientation within the gradient. When the axis of symmetry is resolvable, experiments must confirm that the motor actively aligns its propulsion axis with the chemical field as migration without such alignment indicates passive drift. Conversely, claims of genuine chemotaxis in nanoscale systems necessitate extreme scrutiny. Without an internal mechanism for temporal memory or similar, nanomotors are physically incapable of active steering, making apparent directional motion in these systems overwhelmingly likely to be passive phoretic drift rather than true active swimming behavior.

### Applications for artificial chemotaxis

The concept of the ‘magic bullet’, introduced by Paul Ehrlich in the early 20th century^79^, has profoundly influenced nanomedicine, evolving from passive drug carriers to autonomous systems capable of navigating complex biological environments. Unlike passive vehicles restricted by diffusion and bulk transport, chemotactic nanoparticles utilize chemical gradients to actively penetrate tissue barriers and accumulate precisely at disease sites. While biohybrid systems utilizing cells have been extensively explored, as reviewed by Xia et al.^80^ purely synthetic artificial chemotaxis offers superior design flexibility and scalability. However, it is crucial to note that most instances of application studies deal with phoretic drift-driven “pseudochemotaxis” rather than true chemotaxis. Nevertheless, we highlight notable examples where these synthetic mechanisms, whether relying on phoresis or chemotaxis, are paving the way for breakthroughs in biomedical and technological sectors.

**Fig. 8:**
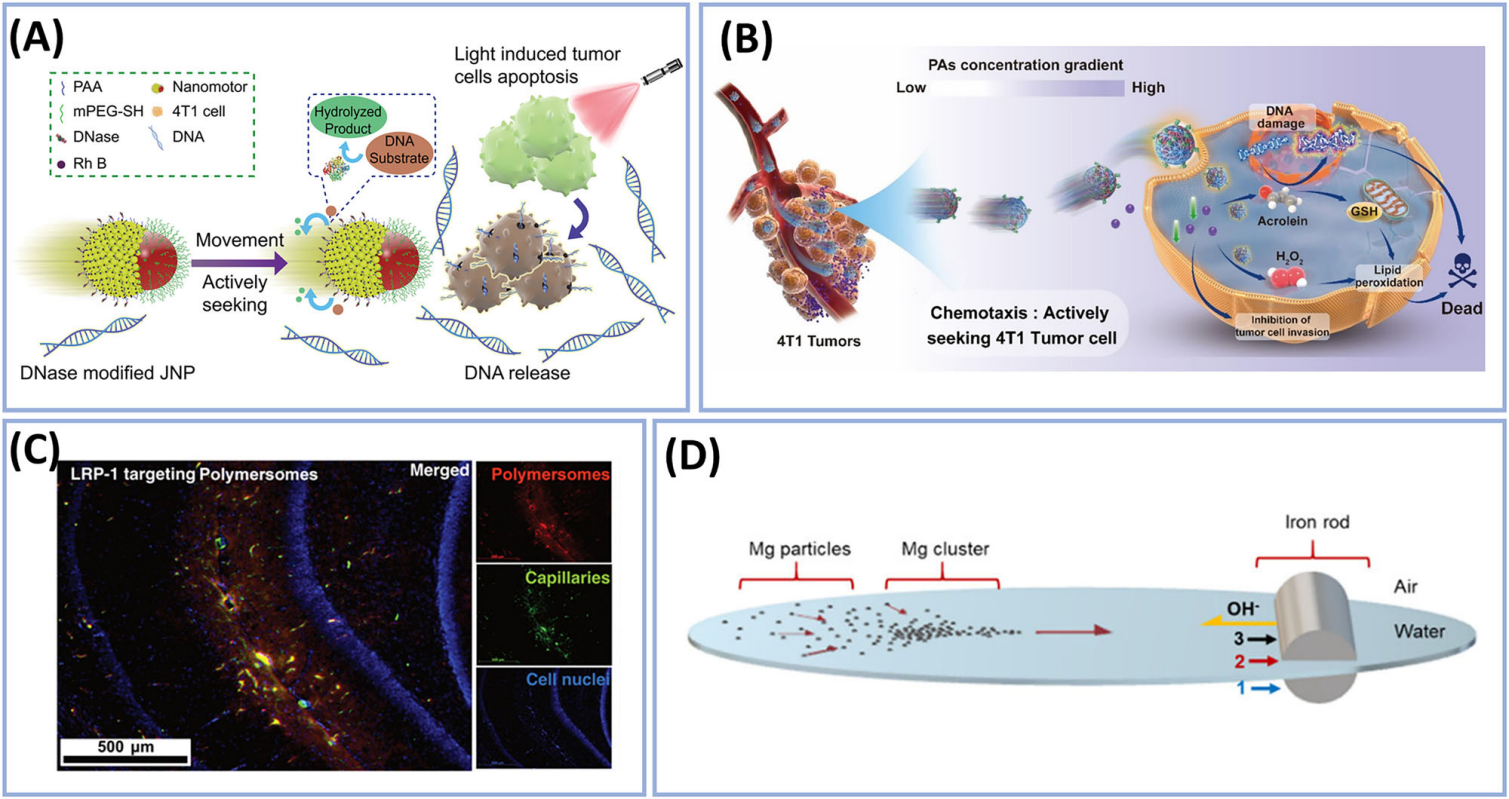
Application of chemotactic micro/nanomotors. **A** Nanomotors activated by apoptotic tumor DNA for targeted cancer therapy. Reprinted with permission from ref. ^35^. Copyright 2021 American Chemical Society. **B** Enzymatic nanomotors exhibiting chemotaxis to enhance product-based cancer treatment. Reprinted with permission from ref. ^44^. Copyright 2025 Elsevier. **C** Chemotactic polymeric vesicles engineered for crossing the blood-brain barrier. Reprinted from ref. ^9^. CC BY 4.0. **D** Chemotaxis of magnesium microparticles for detecting corrosion spots for material remediation. Reprinted with permission from ref. ^81^. Copyright 2021 Wiley-VCH.

In solid tumor therapy, penetrating the dense extracellular matrix remains a critical challenge. Artificial chemotaxis addresses this by exploiting gradients unique to the tumor microenvironment. For instance, specific gradients can be generated, with light induced tumor cell apoptosis, producing apoptotic DNA gradients that have been employed to develop nanomotors capable of seeking cancer cells (Fig. 8A)^35^. Motors can also be used to navigate gradients endogenously expressed by cancer cells, for instance Tan et al. pioneered this by designing nanomotors powered by plasma amine oxidase, which track overexpressed polyamines to deliver cytotoxic agents directly to cancer cells (Fig. 8B)^44^. Similarly, platinum nanoflowers driven by lactate gradients have been engineered to actively penetrate deep tumor tissue, significantly improving therapeutic efficacy compared to passive controls^43^.

Beyond cellular targeting, directional migration is proving essential for crossing physiological barriers. Joseph et al. demonstrated that glucose-powered synthetic vesicles could successfully cross the blood-brain barrier, opening new avenues for treating central nervous system disorders^9^ (Fig. 8C). In ocular therapy, where the dense vitreous humor restricts drug diffusion, Ju et al. ^34^ developed ultrasmall nanomotors engineered with single-atom catalysts. These devices convert endogenous glucose into propulsion, actively navigating the viscous vitreous to deliver antioxidants to the retina. This capability extends to microbial barriers as well. Wu et al. recently introduced swarms that amplify weak acidic signals by over 10^4^ times. These swarms can detect proton gradients from bacterial biofilms and actively penetrate deep dental fissures to eradicate biofilms via the localized release of silver ions^61^. Similarly, in inflammatory diseases, zwitterion-based nanomotors have been shown to track reactive oxygen species and nitric oxide, ensuring the precise delivery of anti-inflammatory agents to affected tissues^39^.

While artificial chemotaxis of micro/nanomotors in biomedical applications dominate the field, the principles are increasingly being applied in industrial engineering. For instance, magnesium micro-swimmers have been designed to function as autonomous repair agents; they utilize pH and ionic gradients to locate corrosion sites in pipelines, docking at the source to form a protective layer (Fig. 8D)^81^. This demonstrates the versatility of chemotactic principles in solving real world problems.

Despite these promising applications, several fundamental limitations remain. Firstly, the specificity of the chemotactic response is currently restricted. Unlike biological cells, which can navigate toward complex signaling molecules independent of their metabolic energy source, the vast majority of synthetic chemotaxis is merely a response to the gradient of the motor’s own fuel or in some cases, towards proton^82,83^ or ionic gradients^81^. This dependence effectively tethers the navigation to the power source, limiting the ability to target arbitrary disease markers that do not double as fuel.

Furthermore, even when a gradient is present, the chemotactic response of synthetic micro/nanomotors is often intrinsically weak. Unlike biological cells, which possess sophisticated internal signaling cascades to amplify minute chemical differences, synthetic motors typically rely on direct phoretic interactions that scale linearly with the gradient. Consequently, they often require steep, high-concentration gradients to overcome Brownian motion and achieve directional drift, limiting their utility in physiological environments where chemical signals are often subtle. To address this, the field is actively shifting toward amplification mechanisms designed to boost sensitivity. A breakthrough in this direction is the development of ferrochemical swarms, which utilize collective interactions^61^.

We anticipate that the discovery of similar amplification mechanisms, whether through collective coupling, autocatalytic cascades, will be crucial for achieving robust chemotactic responses, ultimately unlocking a much broader range of biomedical and industrial applications. Furthermore, another significant limitation arises with chemotactic micro/nanomotors primarily driven by electrolyte diffusiophoresis or electrophoresis. Their motion is inherently limited in complex ionic media, such as blood or body fluids. A promising avenue for future research, therefore, lies in the development of multimodal chemotactic motors. In this approach, motion and directional control can be separated into distinct modalities; for example, propulsion could be powered by magnetic fields or ultrasound to ensure robust movement, while chemotaxis acts solely as the steering mechanism to guide the device toward its target. This evolution from simple phoretic drifters to robust, steered systems will be instrumental in establishing the practical viability of artificial chemotaxis for targeted biomedical and industrial applications.

## Conclusions

The past two decades have witnessed significant developments not only in our understanding of artificial chemotaxis but also in practical applications of artificial chemotaxis in micro/nanomotors. As highlighted before, one of the concerns with the field has to do with the ambiguous definition of chemotaxis and the specific methods utilized to evaluate chemotaxis in micro/nanomotors. Moving forward, we anticipate that the field will coalesce around a stricter definition of chemotaxis that clearly separates it from passive phoretic effects, alongside the widespread adoption of standardized metrics for experimental assessment. The need for complex experimental setups such as the stop-flow setup demonstrates one critical drawback of the current chemotactic micromotors. Artificial chemotaxis, as is, is a relatively weak effect. While carefully designed experiments can demonstrate some degree of directional motion, its strength is vastly inferior to that of biological chemotaxis observed in bacteria and eukaryotic cells, making the realization of effective artificial chemotaxis in micro/nanomotors in complex environments, such as biological fluids, highly unlikely. For the most part, this has to do with the simple and straightforward nature of artificial chemotaxis. Biological chemotaxis, on the other hand, is robust and sensitive to the shallow gradients typically found in biological environments. To design truly chemotactic micro- and nano-scale motors that can go beyond simple directional bias and preferential accumulation, it will be necessary to develop these systems with more complex capabilities in terms of both sensing and responding to weak and shallow gradients. Such developments could enable artificial systems to navigate complex and dynamic environments, bringing them closer to the robustness and adaptability seen in living organisms, and allowing for practical applications.
